# Análise dos desfechos clínicos da microdescompressão lombar na espondilolistese degenerativa de grau 1 e na estenose estável do canal lombar

**DOI:** 10.1055/s-0045-1810037

**Published:** 2025-08-18

**Authors:** Ajaybir Singh

**Affiliations:** 1 Departamento de Ortopedia, Guru Gobind Singh Medical College and Hospital, Baba Farid University of Health Sciences, Faridkot, Punjab, Índia

**Keywords:** ciática, degeneração do disco intervertebral, doenças da coluna vertebral, dor lombar, espondilolistese, intervertebral disc degeneration, low back pain, sciatica, spinal diseases, spondylolisthesis

## Abstract

**Objetivo:**

Avaliar os desfechos clínicos de um procedimento de descompressão sem fusão em pacientes com espondilolistese degenerativa de baixo grau ou estenose estável do canal lombar.

**Métodos:**

O estudo analisou 50 casos de doença degenerativa lombar submetidos à descompressão de um único nível. A avaliação radiográfica dinâmica foi utilizada para determinação de instabilidade. O grupo 1 incluiu pacientes com estenose lombar estável e o grupo 2 incluiu pacientes com espondilolistese degenerativa de grau 1 de Meyerding. Dois anos após a microdescompressão lombar, os desfechos funcionais foram analisados segundo o Índice de Incapacidade de Oswestry (ODI, do inglês Oswestry Disability Index) e a Escala Visual Analógica (EVA) de dor lombar e nos membros inferiores.

**Resultados:**

O grupo 1 incluiu 25 casos com ODI médio de 75,36 ± 13,59 e EVA média de dor lombar de 5,92 ± 3,45 e de dor nos membros inferiores de 8,92 ± 1,81. O grupo 2 incluiu 25 casos com ODI médio de 68,75 ± 11,81 e EVA média de dor lombar de 8 ± 1,22 e de dor nos membros inferiores de 7,87 ± 1,57. Em 2 anos, o ODI médio do grupo 1 melhorou para 22,64 ± 17,2 ( *p* < 0,0001) e a EVA média de dor lombar caiu para 2,04 ± 1,86 ( *p* = 0,0002) e de dor nos membros inferiores diminuiu para 1,56 ± 1,97 ( *p* < 0,0001). O grupo 2 apresentou ODI médio de 24 ± 10,6 ( *p* < 0,0001) e EVA média de dor lombar de 2,12 ± 1,43 ( *p* = 0,0009) e de dor nos membros inferiores de 2,56 ± 1,35 ( *p* = 0,0008). Ambos os grupos apresentaram melhora funcional estatisticamente comparável.

**Conclusão:**

A microdescompressão lombar produziu desfechos funcionais comparáveis em ambos os grupos. Este procedimento é uma opção viável para preservação da integridade do segmento de movimento lombar na espondilolistese degenerativa de baixo grau (grau 1 de Meyerding).

## Abstract (en)

**Objective:**

To assess the clinical outcomes of a non-fusion decompression procedure in degenerative low-grade spondylolisthesis versus stable lumbar canal stenosis.

**Methods:**

The study analyzed 50 cases of lumbar degenerative pathology that underwent decompression involving a single level. Dynamic X-ray assessment was employed to evaluate instability. Group 1 included stable lumbar stenosis, while group 2 included Meyerding grade-1 degenerative spondylolisthesis. Two years postmicrolumbar decompression, the final functional outcomes were assessed using the Oswestry Disability Index (ODI) and the Visual Analog Scale (VAS) for backache and leg pain.

**Results:**

Group 1 included 25 cases with a mean ODI of 75.36 ± 13.59, mean VAS for backache of 5.92 ± 3.45, and mean VAS for leg pain of 8.92 ± 1.81. Group 2 included 25 cases with a mean ODI of 68.75 ± 11.81, mean VAS backache of 8 ± 1.22, and mean VAS leg pain of 7.87 ± 1.57. At 2 years, group 1's mean ODI improved to 22.64 ± 17.2 ( *p* < 0.0001), mean VAS backache reduced to 2.04 ± 1.86 ( *p* = 0.0002), and VAS leg pain reduced to 1.56 ± 1.97 ( *p* < 0.0001). Group 2 showed a mean ODI of 24 ± 10.6 ( *p* < 0.0001), backache mean VAS of 2.12 ± 1.43 ( *p* = 0.0009), and mean VAS leg pain of 2.56 ± 1.35 ( *p* = 0.0008). Both groups showed statistically comparable functional improvement.

**Conclusion:**

Microlumbar decompression yielded comparable functional outcomes in both groups. This procedure presents a viable option for preserving the integrity of lumbar motion segment in degenerative low grade (Meyerding grade 1) spondylolisthesis.

## Introdução

A instabilidade segmentar da coluna lombar decorrente de doenças degenerativas é um problema bem reconhecido. Essa instabilidade geralmente afeta os níveis de L4–5 e de L5–S1, que têm maior amplitude de movimento. Pacientes com degeneração da coluna instável à radiografia tendem a receber a recomendação de estabilização cirúrgica como opção de tratamento. 1 No entanto, a adição de fusão tem seu próprio conjunto de complicações. Logo, uma opção sem fusão para descompressão é desejável em casos de degeneração da coluna lombar. 2

Radiografias, tomografia computadorizada (TC) e ressonância magnética (RM) são ferramentas diagnósticas comumente utilizadas para avaliação de doenças degenerativas lombares. Embora esses métodos sejam inerentemente estáticos, radiografias dinâmicas são cada vez mais empregadas para detecção da instabilidade radiológica. Além disso, a coluna lombar apresenta uma ampla amplitude normal de movimento, o que pode inadvertidamente direcionar o tratamento para a cirurgia de fusão. Essa situação é particularmente pertinente em casos de espondilolistese degenerativa de baixo grau (grau 1 de Meyerding). Vários estudos tentaram definir anomalias de translação, rotação e ângulo em radiografias. 3 4 5 O procedimento de microdescompressão lombar e discectomia minimiza a perda óssea, preserva segmentos móveis e permite a descompressão adequada dos elementos nervosos. 6

## Materiais e Métodos

Este trabalho foi aprovado pelo Comitê de Ética institucional sob o registro n ^o^ ECR/836/lnst./PB/2016/RR-20, n ^o^ IEC/29.

O estudo incluiu 50 pacientes submetidos à cirurgia para tratamento de doença degenerativa da coluna lombar em um único nível. A RM foi utilizada para avaliação da extensão da patologia. Além disso, radiografias pré-operatórias em flexão e extensão da coluna lombar foram realizadas para análise de quaisquer sinais de instabilidade radiológica. As radiografias em perfil de flexão-extensão foram realizadas como descrito por Putto e Tallroth. 4

Para avaliar a estabilidade do segmento móvel, os movimentos translacional e angular foram calculados em radiografias de acordo com Dupuis et al. 5 O acompanhamento clínico pós-operatório foi realizado em 6 semanas, 3 e 6 meses, e 1 e 2 anos.

A avaliação clínica pré e pós-operatória utilizou a Escala Visual Analógica (EVA) para dor lombar e nos membros inferiores e o desfecho funcional foi determinado pelo Índice de Incapacidade de Oswestry (ODI, do inglês Oswestry Disability Index).

Os pacientes incluídos eram adultos (maiores de 18 anos) submetidos à cirurgia de coluna em um único nível para tratamento de estenose degenerativa do canal lombar, prolapso de disco intervertebral e espondilolistese degenerativa de baixo grau (grau 1 de Meyerding).

Os critérios de exclusão foram definidos como casos de instabilidade da coluna por fraturas vertebrais, tumores espinhais, espondilolistese degenerativa de grau 2 de Meyerding ou superior, infecções espinhais, listese lítica, espondilólise, necessidade de cirurgia de revisão da coluna lombar, acometimento associado do segmento cervical ou torácico com necessidade de intervenção cirúrgica e doenças intradurais. O comitê de ética aprovou a realização deste estudo.

### Técnica Cirúrgica

A microdisectomia lombar foi realizada com o paciente em decúbito ventral sob anestesia geral. O nível de descompressão foi marcado por fluoroscopia. Foi utilizada uma abordagem mediana à coluna lombar posterior. Após a dissecção subperióstea, foi realizada a laminotomia microscopicamente assistida com broca e pinça de Kerrison. O ligamento amarelo hipertrófico foi removido para descompressão do canal central. A descompressão do recesso lateral, caso necessária, foi feita com uma secção parcial da faceta. Em todos os pacientes, a raiz nervosa foi visualizada e adequadamente descomprimida. O procedimento de discectomia ocorreu após a anulotomia. O fechamento foi realizado por planos.

A movimentação dos pacientes foi permitida no dia seguinte à cirurgia. Exercícios de fortalecimento da coluna e da pelve foram iniciados conforme a tolerância dos indivíduos. A fisioterapia e a reabilitação foram intensificadas gradativamente de acordo com a melhora clínica dos pacientes.

## Resultados

### Dados Demográficos e Características Basais dos Pacientes

Cinquenta e três pacientes participaram do estudo, mas três foram perdidos durante o acompanhamento. O grupo 1 foi composto por 25 casos com estabilidade radiológica do segmento lombar e o grupo 2 foi formado por 25 pacientes com espondilolistese degenerativa de baixo grau (grau 1 de Meyerding) de acordo com a avaliação por meio de radiografias dinâmicas. Todos os pacientes foram submetidos à microdescompressão mediana de um único nível. As avaliações clínicas foram realizadas aos 3 e 6 meses, e 1 e 2 anos após a cirurgia. Os desfechos clínicos de ambos os grupos foram analisados por EVA e ODI 2 anos após a cirurgia.

### Distribuição Etária e Sexual

A Tabela 1 mostra a distribuição etária de ambos os grupos. Os grupos 1 e 2 tinham 25 pacientes cada, com distribuição etária comparável ( *p* = 0,6808 e *p* > 0,05, respectivamente, de acordo com o teste exato de Fisher). A Tabela 2 resume a distribuição por sexo: o grupo 1 incluiu 12 homens e 13 mulheres, enquanto o grupo 2 tinha 14 homens e 11 mulheres. A análise estatística não demonstrou diferença significativa na distribuição por sexo entre os 2 grupos ( *p* = 0,6911, teste exato de Fisher).

**Tabela 1:** Distribuição etária dos pacientes dos grupos 1 e 2

Idade (anos)	Número (%)	
	Grupo 1	Grupo 2
≤ 30	3	2
31–40	4	7
41–50	10	2
51–60	6	10
≥ 61	2	4
Total	25	25

**Nota** : Valor de *p* = 0,6808 segundo o teste exato de Fisher, com limite de idade de 50 anos.

**Tabela 2:** Distribuição dos pacientes por sexo nos grupos 1 e 2

Sexo	Número (%)	
	Grupo 1	Grupo 2
Masculino	12	14
Feminino	13	11
Total	25	25

**Nota** : Valor de *p* = 0,6911 segundo o teste exato de Fisher.

### Segmento Lombar Acometido

A Tabela 3 descreve a distribuição do acometimento do segmento lombar. No grupo 1, a maioria dos casos apresentava comprometimento do segmento L4 a L5 (14 casos), seguido por L5 a S1 (8 casos), L3 a L4 (2 casos) e L2 a L3 (1 caso). No grupo 2, o acometimento ocorreu principalmente em L4 a L5 (17 casos) e L5 a S1 (6 casos), com casos isolados em L2 a L3 e L3 a L4.

**Tabela 3:** Distribuição dos pacientes por nível acometido nos grupos 1 e 2

Diagnóstico	Número	
	Grupo 1	Grupo 2
L2–L3	1	1
L3–L4	2	1
L4– L5	14	17
L5–S1	8	6

### Quadro Clínico

A Tabela 4 mostra as queixas principais dos pacientes. No grupo 1, todos os 25 pacientes relataram radiculopatia e 19 também apresentavam dor lombar e irradiação da dor para os membros inferiores. Da mesma forma, todos os 25 pacientes do grupo 2 se queixaram de radiculopatia e 21 pacientes também tinham dor lombar axial. A distância de claudicação também foi registrada, revelando que 33 pacientes apresentavam claudicação a ≤ 50 m, sete a 100 m e 10 pacientes a 200 m.

**Tabela 4:** Distribuição das queixas principais nos grupos 1 e 2

Queixas	Número (%)	
	Grupo 1 N = 25	Grupo 2 N = 25
Dor nas costas	19 (76%)	21 (84%)
Radiculopatia	25 (100%)	25 (100%)

### Desfechos Clínicos

A Tabela 5 apresenta os desfechos clínicos do grupo 1. A pontuação média de ODI melhorou significativamente de 75,36 ± 13,59 no período pré-operatório para 22,64 ± 17,2 2 anos após a cirurgia ( *p* < 0,0001). As pontuações de EVA para dor nas costas e nos membros inferiores também melhoraram de forma significativa. A EVA média de dor nas costas caiu de 5,92 ± 3,45 no período pré-operatório para 2,04 ± 1,86 ( *p* = 0,0002), enquanto a EVA média de dor nos membros inferiores diminuiu de 8,9 para 1,56 ( *p* < 0,0001).

**Tabela 5:** Comparação do ODI e da EVA de dor nas costas e nos membros inferiores antes da cirurgia e aos 2 anos de acompanhamento no grupo 1

Grupo	Grupo 1								Valor de *p* *
	Antes da cirurgia				Após a cirurgia (em 2 anos)				
Medidas estatísticas	N	Média	DP	Intervalo	N	Média	DP	Intervalo	
ODI	25	75.36	13.59	50–96	25	22.64	17.2	0–54	< 0.0001
EVA de dor nas costas	25	5.92	3.45	0–10	25	2.04	1.86	0–5	0.0002
EVA de dor nos membros inferiores	25	8.92	1.61	2.50–10	25	1.56	1.97	0–5	< 0.0001

**Abreviaturas** : DP, desvio padrão; EVA, Escala Visual Analógica; ODI, Oswestry Disability Index (Índice de Incapacidade de Oswestry).

**Nota** : *Teste dos postos sinalizados de Wilcoxon.

A Tabela 6 resume os desfechos clínicos do grupo 2. O ODI médio melhorou de 68,75 ± 11,81 no período pré-operatório para 24 ± 10,6 2 anos após a cirurgia ( *p* < 0,0001). A EVA média de dor nas costas diminuiu significativamente de 8 ± 1,22 para 2,12 ± 1,43 ( *p* = 0,0009). Além disso, a EVA média de dor nos membros inferiores melhorou de 7,87 no pré-operatório para 2,56 2 anos após a cirurgia ( *p* = 0,0008).

**Tabela 6:** Comparação do ODI, EVA de dor nas costas e nos membros inferiores antes da cirurgia e aos 2 anos de acompanhamento no grupo 2

Grupo	Grupo 2								Valor de *p*
	Antes da cirurgia				Após a cirurgia (em 2 anos)				
Parâmetros estatísticos	N	Média	DP	Intervalo	N	Média	DP	Intervalo	
ODI	25	68.75	11.81	46–84	25	24	10.6	10–38	< 0.0001
EVA de dor nas costas	25	8	1.22	6–10	25	2.12	1.43	0–4	0.0009
EVA de dor nos membros inferiores	25	7.87	1.57	5–9	25	2.56	1.35	0–5	0.0008

**Abreviaturas** : DP, desvio padrão; EVA, Escala Visual Analógica; ODI, Oswestry Disability Index (Índice de Incapacidade de Oswestry).

**Nota** : *Teste dos postos sinalizados de Wilcoxon.

A Tabela 7 apresenta os resultados comparativos entre os dois grupos aos dois anos de acompanhamento. O ODI médio do grupo 1 (22,64 ± 17,2) foi estatisticamente comparável ao do grupo 2 (24 ± 10,6), com valor de *p* de 0,7924. Da mesma forma, as pontuações de EVA de dor nas costas (2,04 ± 1,86) no grupo 1 em comparação ao grupo 2 (2,12 ± 1,43; *p* = 0,7154) e dor nos membros inferiores (1,56 no grupo 1 e 2,56 no grupo 2; *p* = 0,0945) não apresentaram diferenças significativas.

**Tabela 7:** Comparação de ODI e EVA de dor nas costas e nos membros inferiores nos grupos 1 e 2 aos 2 anos de acompanhamento

Condição	Aos 2 anos de acompanhamento								Valor de *p* *
Grupo	Grupo 1				Grupo 2				
Parâmetros estatísticos	N	Média	DP	Intervalo	N	Média	DP	Intervalo	
ODI	25	22.64	17.2	0–54	25	24	10.6	10–38	0.7924
EVA de dor nas costas	25	2.04	1.86	0–5	25	2.12	1.43	0–4	0.7154
EVA de dor nos membros inferiores	25	1.56	1.97	0–5	25	2.56	1.35	0–5	0.0945

**Abreviaturas** : DP, desvio padrão; EVA, Escala Visual Analógica; ODI, Oswestry Disability Index (Índice de Incapacidade de Oswestry).

**Nota** : *Teste da soma dos postos de Wilcoxon.

### Acompanhamento

No último acompanhamento, 34 pacientes não relataram sintomas de claudicação. Dezesseis pacientes apresentaram sintomas mínimos, como peso ou dormência branda nos membros inferiores, mas conseguiam caminhar distâncias superiores a um quilômetro com o tratamento conservativo dos sintomas.

## Discussão

O processo de instabilidade degenerativa se desenvolve em três fases distintas: disfunção, instabilidade e reestabilização. A instabilidade segmentar é causada pela degeneração das facetas e dos discos, levando à frouxidão e à movimentação anormal sob a carga fisiológica das atividades diárias. 7 Panjabi 8 propôs um conceito trimodal de estabilidade espinhal. As manifestações clínicas da instabilidade segmentar são caracterizadas por sintomas inespecíficos, incluindo dor lombar acompanhada por dor radicular, particularmente durante mudanças na postura.

Um estudo recente de Van Grafhorst et al., 9 de 2025, relatou os desfechos obtidos em 9 anos de acompanhamento após a descompressão sem fusão em pacientes com estenose espinhal lombar acompanhada ou não por espondilolistese degenerativa de baixo grau (I). Os 250 casos com espondilolistese de baixo grau apresentaram taxa de satisfação de 69%, comparável aos 200 casos do grupo com estenose degenerativa (68%). A descompressão isolada produziu desfechos duradouros e satisfatórios, com taxas de reoperação baixas, de 7% no grupo com espondilolistese e 6% no grupo com estenose.

Em 2023, Abdel-Fattah et al. 10 publicaram uma revisão sistemática e meta-análise de 6 ensaios clínicos randomizados (ECRs) em 2023 com um total de 531 pacientes com idade média de 66,2 anos e acompanhamento médio de 27,4 meses. O estudo demonstrou que a descompressão isolada (DA) não é inferior à descompressão com fusão (DF) em termos de dor, incapacidade ou taxas de reoperação em pacientes idosos com estenose espinhal e espondilolistese degenerativa de baixo grau. A DA está associada a menos complicações cirúrgicas, menor tempo operatório e menor carga perioperatória. Portanto, as decisões clínicas devem ponderar os riscos cirúrgicos, as comorbidades do paciente e os objetivos funcionais.

Liu et al. 6 conduziram um estudo de modelo de elementos finitos para avaliação do impacto biomecânico das descompressões na estabilidade lombar. O estudo comparou segmentos lombares normais àqueles com espondilolistese de grau 1, cada um em três cenários cirúrgicos: hemilaminectomia, laminectomia total e facetectomia parcial. A hemilaminectomia e a facetectomia parcial foram biomecanicamente seguras na espondilolistese de baixo grau, mantendo a estabilidade do segmento e provocando alterações mínimas na mecânica espinhal. No entanto, a laminectomia total alterou significativamente a biomecânica e deve ser usada com cautela na espondilolistese devido ao aumento do movimento, da pressão discal e do estresse nos elementos posteriores. O modelo de elementos finitos apoiou estratégias minimamente invasivas que preservam a estabilidade e, ao mesmo tempo, levam à descompressão.

Um ECR multicêntrico, chamado Swedish Spinal Stenosis Study, publicado em 2024 por Karlsson et al., 11 mostrou os desfechos em longo prazo (5 anos) da DA isolada e da DF em pacientes com estenose espinhal lombar acompanhada ou não por espondilolistese degenerativa. De 247 pacientes com idade entre 50 e 80 anos, 124 foram incluídos no grupo DA e 123 no grupo DF. Os escores de avaliação de qualidade de vida ODI, EVA e Qualidade de Vida Europeia-5 Dimensões (European Quality of Life-5 Dimensions, EQ-5D) mostraram que a fusão não gera desfechos clínicos superiores, pois a qualidade de vida e o alívio da dor nos membros inferiores foram melhores em pacientes submetidos à DA aos 5 anos de acompanhamento. A descompressão sozinha é preferível por levar a desfechos semelhantes ou melhores e ter menores riscos de complicações, tempo de hospitalização e menos problemas no segmento adjacente. As taxas de reoperações foram semelhantes em ambos os grupos, mas tiveram causas diferentes, sendo de 24% no grupo DF (principalmente por estenose de nível adjacente) e 22% no grupo DA (principalmente por reestenose).

Kgomotso et al., 12 em 2024, tiveram como objetivo avaliar a eficácia em longo prazo da descompressão isolada em comparação à descompressão com fusão instrumentada em pacientes com espondilolistese lombar degenerativa com acompanhamento por cinco anos. O estudo foi estruturado como um ECR multicêntrico e de não inferioridade (NORDSTEN-DS) para determinar se a DA é não-inferior à DF. O estudo incluiu 267 pacientes com idades entre 18 e 80 anos com estenose lombar e espondilolistese de pelo menos 3 mm no nível estenótico. Os casos foram randomizados para descompressão isolada ( *n* = 134) ou descompressão com fusão ( *n* = 133). Os autores concluíram que a DA não é inferior à DF instrumentada para o tratamento de espondilolistese lombar degenerativa em 5 anos, sem diferenças significativas nos desfechos clínicos ou nas taxas de reoperação.

Um estudo recente de Unterfrauner et al. 13 teve como objetivo comparar a eficácia da DA e da DF em pacientes com espondilolistese degenerativa lombar e estenose espinhal ao longo de um período de acompanhamento de 3 anos usando dados do Lumbar Stenosis Outcome Study (LSOS). Os autores empregaram uma emulação de ensaio-alvo para comparação com o ECR NORDSTEN-DS para melhorar a inferência causal em dados observacionais. O estudo concluiu que a DA deve ser considerada a principal opção cirúrgica em pacientes com espondilolistese degenerativa lombar e estenose espinhal, visto que a fusão não trouxe mais benefícios em termos de qualidade de vida relacionada à saúde, redução da dor ou satisfação em 3 anos e aumentou as necessidades de fisioterapia. Esse achado desafia o uso rotineiro da fusão nesses casos, sugerindo que a descompressão isolada é eficaz e demanda menos recursos.

A revisão sistemática e meta-análise de 3 ECRs e 9 estudos de coorte publicada por Cheng et al. 14 em 2024 teve como objetivo a avaliação da eficácia e da segurança da descompressão isolada em comparação à descompressão com fusão em pacientes com estenose espinhal lombar em um único nível e espondilolistese. A análise incluiu 6.182 pacientes, sendo 2.339 no grupo de descompressão isolada e 3.783 no grupo de descompressão com fusão. Os resultados indicaram que a descompressão isolada não é inferior à descompressão com fusão em pacientes com estenose espinhal lombar em um único nível e espondilolistese. Devido ao menor tempo operatório, a menor perda sanguínea intraoperatória e a ausência de aumento nas taxas de complicações e reoperação, a descompressão isolada é recomendada como a principal opção cirúrgica.

Um estudo de coorte retrospectiva de Van Grafhorst et al. 2 teve como objetivo avaliar a eficácia da descompressão isolada como tratamento da estenose lombar sintomática com espondilolistese degenerativa de baixo grau e a incidência e os desfechos da reoperação com ou sem fusão instrumentada subsequente. Um total de 934 pacientes foram submetidos à cirurgia para tratamento da estenose espinhal lombar, incluindo 253 pacientes com espondilolistese degenerativa. Todos os pacientes foram primeiramente submetidos à descompressão isolada; apenas três pacientes com espondilolistese foram submetidos à descompressão primária com fusão. Depois deste primeiro procedimento, 80% dos pacientes com estenose e 74% dos pacientes com espondilolistese relataram satisfação ( *p* = 0,059). As taxas de reoperação foram de 12% no grupo com estenose (1,2% foram submetidos à fusão) e 17% no grupo com espondilolistese (38,1% foram submetidos à fusão). O estudo concluiu que a descompressão isolada é um tratamento eficaz para a estenose lombar sintomática com espondilolistese degenerativa de baixo grau. Nas reoperações, a descompressão secundária, às vezes com ressecção estendida do arco superior, geralmente foi suficiente. A fusão foi necessária em alguns casos, reforçando o argumento de que a maioria dos pacientes não precisa de fusão instrumentada de rotina.

O estudo de Khashan et al. 15 teve como objetivo avaliar os desfechos clínicos e as taxas de complicações e reoperação da descompressão tubular minimamente invasiva (MI) para tratamento da estenose espinhal lombar com ou sem espondilolistese degenerativa de baixo grau estável. Os autores examinaram especificamente se a presença de espondilolistese estável comprometia os desfechos da descompressão MI. Os 96 pacientes incluíam 53 indivíduos com espondilolistese degenerativa estável de baixo grau e 43 pacientes com estenose espinhal. A presença de espondilolistese estável não comprometeu os desfechos clínicos ou aumentou as taxas de complicações ou reoperação. O estudo concluiu que a descompressão tubular MI é um procedimento eficaz e seguro para pacientes com estenose espinhal lombar, independentemente da presença de espondilolistese degenerativa estável de baixo grau.

Ghiselli et al. 16 conduziram um estudo com 215 casos de fusão lombar posterior e observaram uma prevalência de degeneração sintomática do segmento adjacente com necessidade de descompressão ou artrodese. O estudo previu uma incidência de 16,5% de degeneração sintomática do segmento adjacente em 5 anos e uma incidência de 36,1% em dez anos. Carreon et al. 17 observaram que a descompressão com fusão em indivíduos com mais de 65 anos estava associada a uma maior incidência de complicações graves, como infecções do sítio cirúrgico (prevalência de 10%) e complicações menores, incluindo infecções do trato urinário (prevalência de 34%). Além disso, o aumento dos níveis de fusão levou a uma elevação concomitante na perda sanguínea e no tempo operatório.

Nossos achados condizem com as conclusões desses estudos, que questionaram a importância da fusão e defenderam uma perspectiva favorável à não fusão e a uma abordagem de descompressão menos invasiva.

No entanto, nosso estudo apresenta algumas limitações, como sua natureza retrospectiva, que restringe a generalização de seus achados. Embora radiografias dinâmicas de flexão-extensão da coluna lombar sejam comumente empregadas na tomada de decisão em casos de artrodese lombar, parâmetros radiográficos, como a avaliação do equilíbrio sagital global, são cada vez mais utilizados. A ausência de um grupo de artrodese é outra limitação, visto que os resultados apresentados são apenas de uma cirurgia sem artrodese.

## Conclusão

A coluna lombar suporta mais o peso corporal do que outras regiões da coluna, facilitando uma ampla gama de movimentos de flexão e extensão. Consequentemente, a preservação do movimento lombar é importante. Procedimentos cirúrgicos que visam preservar o movimento da coluna lombar têm recebido atenção significativa e abrangem diversas técnicas. É crucial identificar a população de pacientes que pode se beneficiar ao evitar um procedimento de fusão. Em nosso estudo, uma análise comparativa da espondilolistese degenerativa de baixo grau à estenose estável do canal lombar submetida a um procedimento cirúrgico semelhante sem fusão demonstrou desfechos clínicos comparáveis. Consequentemente, a microdescompressão lombar surge como uma opção de tratamento viável para preservar o segmento de movimento lombar na espondilolistese degenerativa de baixo grau.
